# Development of Simple and Advanced Adult Proximal Tibia Simulators for a Decentralized Simulation-Based Education Model to Teach Paramedics-in-Training the Intraosseous Infusion Procedure

**DOI:** 10.7759/cureus.30929

**Published:** 2022-10-31

**Authors:** Mithusa Sivanathan, Luz Yanguez Franco, Shitij Joshi, Julia Micallef, Dale Button, Adam Dubrowski

**Affiliations:** 1 Health Sciences, Ontario Tech University, Oshawa, CAN; 2 Engineering and Applied Science, Ontario Tech University, Oshawa, CAN; 3 Paramedicine, Durham College, Oshawa, CAN

**Keywords:** pediatric emergency medicine, pediatrics, emergency medicine, additive manufacturing, three-dimensional printing, simulation-based medical education, intraosseous infusion, simulator, training, 3d-printing

## Abstract

Introduction

During the COVID-19 pandemic, public health had advised practicing social distancing which led to the temporary shutdown of simulation laboratories or centralized simulation-based education model, shared spaces that healthcare workers such as paramedics use to train on important hands-on clinical skills for the job. One such skill is intraosseous (IO) access and infusion, the delivery of fluids and medication through the marrow or medullary cavity of the bone which provides fast and direct entry into the central venous system. This skill is critical in emergencies when peripheral access is not immediately available. To continue the training of paramedics in life-saving skills like IO infusion in the post-pandemic era, a decentralized simulation-based education (De-SBE) model was proposed. The De-SBE relies on the availability of inexpensive and flexible simulators that can be used by learners outside of the simulation laboratory. However, to date, there is a paucity of simulation design methods that stimulate creativity and ideation, and at the same time, provide evidence of validity for these simulators. Our exploratory research aimed to test a novel approach that combines components of development-related constraints, ideation, and consensus (CIC) approach to develop and provide content validity for simulators to be used in a De-SBE model.

Materials and methods

The development of the IO simulators was constrained to follow a design-to-cost approach. First, a modified design thinking session was conducted with three informants from paramedicine and medicine to gather ideas for the development of two IO simulators (simple and advanced). Next, to sort through, refine, and generate early evidence of the content validity of the simulators, the initial ideas were integrated into a two-round, modified Delphi process driven by seven informants from paramedicine and medicine. In addition, we surveyed the participants on how well they liked the CIC approach.

Results

The CIC approach generated a list of mandatory and optional features that could be added to the IO simulators. Specifically, six features (one mandatory and four optional) for the existing simple IO simulator and eight (three mandatories and five optional) for the advanced IO simulators were identified. Following a design-to-cost approach, the features classified as mandatory for the simple and advanced IO simulators were integrated into the final designs to maintain the feasibility of production for training purposes. The surveys with the participants showed that the CIC approach worked well in the group setting by allowing for various perspectives to be shared freely and ending with a list of features for IO simulator designs that could be used in the future. Some improvements to the approach included flagging for potential users to determine what works best concerning the mode of delivery (online or in person), and duration of the stages to allow for more idea generation.

Conclusion

The CIC approach led to the manufacturing of simple and advanced IO simulators that would suit a training plan catered to teach the IO access and infusion procedure decentrally to paramedics-in-training. Specifically, they have been designed in a manner that allows them to be made easily accessible to the trainees i.e., low costs and high mobility, and work cohesively with online learning management systems which further facilitates the use of a De-SBE model.

## Introduction

During the COVID-19 pandemic, to keep people safe from the infectious disease, public health entities advised social distancing [1]. Unfortunately, this practice led to unintended consequences in the training of healthcare workers [2]. For example, paramedics-in-training could no longer train in simulation laboratories, spaces where they congregated with educators to learn and teach essential psychomotor skills such as intraosseous (IO) access and infusion. Since simulation laboratories, referred to here as a centralized simulation-based education (Ce-SBE) model, were no longer appropriate during the COVID-19 pandemic, an alternative model, known as a decentralized simulation-based education (De-SBE) model, was needed to continue the training of healthcare workers. Decentralized simulation-based education refers to a model where learners can practice clinical, hands-on skills outside of the simulation lab such as in their own homes or other locations that allow for social distancing. The need for this model was specifically heightened during the COVID-19 pandemic as increasing numbers of hospitalized cases and fatalities due to the virus demanded support far greater than the number of trained healthcare workers available [3]. The development of a De-SBE model would potentially lead to uninterrupted training, reduced training costs, and offer students a unique opportunity to customize their learning according to their own needs and pace, which is called mastery-based learning [4]. 

In the post-pandemic era, the De-SBE model can help us optimize the Ce-SBE model to train paramedics-in-training (and other healthcare professionals). For example, current mastery-based simulation programs cannot be offered to students due to human (i.e., trainers) and equipment (i.e., simulators) resource constraints. It is believed that some of these potential benefits of the proposed De-SBE model would be retained in the post-pandemic landscape to complement the traditional ways of training [5]. 

The key elements of De-SBE are the availability of inexpensive and flexible simulators [2], instructions, motivation, and feedback for remote trainees [5]. The current report deals specifically with the methods underpinning the design of inexpensive and flexible simulators for De-SBE. The work is situated in the initial phases of the design-based research methodology [6]. Because the design of these simulators requires communication between designers, clinical educators, and simulation technologists the methodology underpinning the development must be accepted by all stakeholders. In particular, the designers favor methods, such as design thinking [7] that emphasize the generation of ideas and prototypes. On the contrary, clinical educators are mostly concerned with the content validity of the simulators (content validity is defined as the representation of currently available knowledge on a construct of interest [8]). Finally, simulation technologists are mostly concerned with the costs of simulation. Here, we propose a hybrid methodology that combines elements of crowdsourcing of ideas/solutions with a methodology that generates evidence of validity by leveraging consensus-building methods, and finally, puts financial constraints on the design process by using design-to-cost approaches. 

Therefore, this technical report aims to describe this hybrid methodology exemplifying how it works in the context of the development of IO simulators for paramedic training and assess the acceptability of this hybrid method by participating groups of stakeholders. 

## Materials and methods

Educational context

Intraosseous access and infusion refer to delivering fluids and medication through the marrow or medullary cavity of the bone. The bone offers a non-collapsible, direct entry into the central venous system [9]. The IO access and infusion are typically executed if peripheral access, such as intravenous (IV), is not immediately available [10]. The IO access and infusion route allows for rapid and efficacious administration of drugs and fluids which is essential during emergencies [11]. The ability to quickly and effectively obtain IO access and initiate infusions is an important skill for paramedics.

The proposed De-SBE model focuses on teaching paramedics-in-training the IO access and infusion procedure done on the proximal tibia of an adult patient. The elements that make up the De-SBE model will include a learning management system (LMS) designed to develop psychomotor skills remotely using video assessments [5]. In accompaniment to the LMS, 3D-printed and silicone-based adult proximal tibial IO access and infusion simulators, one being simple and the other being advanced, will be used (henceforth referred to as simple and advanced IO simulators going forward). Both IO simulators are being explored based on the earlier work of the development team that demonstrates clinical skills and are better transferred when students practice on simulators of increasing fidelity [12], and the concept of the optimal challenge point which states that the learner should be challenged according to their skill level to yield better long-term performance (i.e., a junior learner would benefit from the simple IO simulator while a senior learner would benefit from the advanced IO simulator) [13].

Procedure

Based on the Tri-Council Policy Statement: Ethical Conduct for Research Involving Humans - TCPS 2, Article 6.11, this work was qualified as a research tool and protocol development [14]. The local Research Ethics Board granted an exemption and issued approval (approval no.: 241-2122). 

The simple and advanced IO models in the scope of this project were previously created by the development team and considered good educational tools requiring a few minor adjustments by clinicians and medical trainees [15,16]. Based on this feedback, to develop these existing IO simulators further yet appropriately for the De-SBE model, a process was required that could facilitate ideation while providing content validity. To fulfill this need, a modified design thinking process to generate ideas followed by a modified Delphi methodology to gather content validity of those ideas was conducted with a group of experts in the IO infusion and access skill to re-develop the IO simulators for the De-SBE model. 

The Constraints-Ideation-Consensus (CIC) Approach

The CIC approach consists of three major methodological approaches. First, after the initial needs have been articulated, a set of constraints need to be put on the design process. Typically, these are related to design-to-cost and design-to-value. Design-to-cost refers to the optimization of the total costs of a product through its design which in the context of simulation can be through sacrificing the realism as demonstrated by our development team through the creation of affordable take-home simulators [2]. Design-to-value, on the other hand, is making sure to deliver the best possible value to the endpoint user through the product without much regard for the costs [17]. 

**Figure 1:**
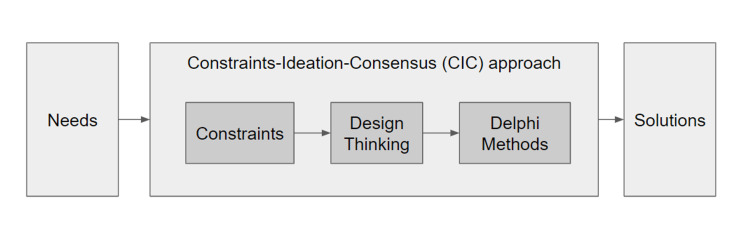
The CIC approach CIC: Constraints-ideation-consensus

Next, once these constraints have been identified, a design thinking process can be used in the ideation phase. Design thinking originates from engineering and business fields, and it is an iterative and non-linear process used to generate innovative solutions creatively and collaboratively with a team that could be multidisciplinary. It comprises five phases: empathizing, defining, ideating, prototyping, and designing [7]. However, in the context of healthcare professionals' education, all educational interventions need to be evidence-based. More specifically, when simulators are built, at a minimum they must show evidence of content validity to ensure that they can serve as educational tools [18]. One way to gather this evidence is by systematic documentation of content expert opinions about the validity of these simulators to serve educational purposes [19]. However, the design thinking process does not provide this type of evidence. Hence, we propose that only the first three phases (empathize, ideate, and define) of the design thinking process be used with a group of individuals, hereafter referred to as informants, to generate the ideas. Next, we propose to replace the last two phases, prototype, and test, with a Delphi method to harness the expert consensus in a fashion that is acceptable to clinical educators. Therefore, to further clarify and gather evidence regarding the content validity of the solutions, a Delphi methodology was used. Delphi methodology is a structured technique used to determine consensus among informants on a solution to a complex problem. In this context, the Delphi methodology was modified as the informants were provided the solutions from the modified design thinking process for their input and to converge on, rather than starting with open-ended questions to solicit solutions [20]. Collectively, this hybrid approach to development will be referred to as the CIC approach (Figure 1).

To test the CIC approach, we selected the development of two IO simulators. We assessed the products as well as surveyed participants who helped with the development to express their opinions about the acceptability of the CIC approach.

Participants

In total, seven participants (i.e., designers, practicing paramedics, and educators) took part in the development process. Three of the seven were involved in the modified design thinking process and all seven informants participated in the modified Delphi methodology. Six were paramedics of which three were from the Region of Durham Paramedic Services (Oshawa, Ontario, CA), one from Durham College (Oshawa, Ontario, CA), one from Lakeridge Health Hospital (Oshawa, Ontario, CA), one from the University of Toronto (Toronto, Ontario, CA), and one from Sunnybrook Hospital (Oshawa, Ontario, CA). One was a medical doctor and was affiliated with Madrecor Hospital (Brazil), Memorial University of Newfoundland (Newfoundland, Ontario, CA), and Ontario Tech University (Oshawa, Ontario, CA). All seven had experience performing the IO access and infusion procedure in the proximal tibia in adult patients. Furthermore, the design process was facilitated by a designer and a simulation expert from Ontario Tech University. 

Constraints

A design-to-cost approach served as a constraint for the development process to create affordable IO simulators under $15 for a simple version and $100 for an advanced version. Note that these are production costs and do not incorporate design-related costs.

Modified Design Thinking Process

The modified design thinking process ran as a two-hour video conference call through Google Meet (Google Inc., Mountain View, California, USA) and comprised two paramedics and one medical doctor. During the modified design thinking process, the informants were asked to 1) empathize with the designers and understand the need to improve the two IO simulators, 2) clearly define the problem, and 3) brainstorm ideas that would address the need and solve the problem. During the empathize phase, the informants were given the learning objectives of the IO simulators: the simple IO model is to be redesigned, if necessary, to teach the epitome of the IO skill, and the advanced IO model, would be updated to incorporate additional elements that would make the simple IO model more challenging. All of the ideas during the modified design thinking process were captured on a Google Jamboard.

Modified Delphi Methodology and Analysis

The ideas that resulted from the modified design thinking session were carried over into a modified Delphi methodology to gather expert consensus on the ideas for improvements to be made to the simple and advanced IO simulators. Seven informants participated in the modified Delphi rounds. The activity was hosted in Google Forms and completed by the informants remotely over two weeks. In the first round, the informants were asked to rank the ideas from the modified design thinking process based on the importance of including the ideas in the final design of the simple and advanced IO simulators using a Likert scale of one to five, where one was considered very unimportant and five was considered very important. Ideas that received an average rating of 3.5 or above were considered important to include while ideas that received an average rating below 3.5 were considered unimportant to include in the final design of the simple and advanced IO simulators [21]. Variability in responses was examined using standard deviation (SD), and ideas that had an SD of 1 or greater were considered for the subsequent modified Delphi round as they indicated low levels of agreement amongst informants on the importance of including the idea in the final design of the simple and advanced IO simulators [21]. To help with the interpretation of the SD values, comments provided through open-field text boxes after every idea were also assessed. Based on this algorithm, ideas requiring further clarification from the informants due to lack of consensus were rephrased and reinstated in the second modified Delphi round. However, the majority of the ideas in the second modified Delphi round were structured as multiple-choice questions, requesting informants to classify the ideas as “mandatory”, “optional/nice-to-have”, or “not required”. This approach was followed because most of the comments provided by informants in the first modified Delphi round naturally offered a similar classification of the ideas using terms such as “mandatory”, “optional/nice-to-have”, or “not required”. Therefore, this format was used to confirm the inclusion or exclusion of tangible ideas into the simple and advanced IO simulators. Abstract ideas that could not be physically represented in the simple and advanced IO simulators that needed revisiting in the second modified Delphi round followed the Likert scale layout used in the first modified Delphi round. The modified Delphi methodology was concluded in two rounds.

## Results

Results of the modified design thinking process

During the modified design thinking process, 14 ideas from three informants were offered on how to improve the simple and advanced IO simulators. The ideas noted in the Google Jamboard from the modified design thinking process have been presented in Table 1. These 14 ideas were used as the starting point for the modified Delphi methodology.

**Table 1:** Ideas generated from the modified design thinking process with three informants IO: Intraosseous, IV: Intravenous

Simple IO Simulator	Advanced IO Simulator
Provides resistance when performing the IO technique on it	Bends and laterally exposes the tibia (e.g., 360-degree swivel on the superior end so that we can also move the leg and position it on the table)
Simulates the bone marrow (e.g., in the hollow space of the bone, place a sponge and add liquid to it)	Made longer from the knee down
Demonstrates people of different weights (i.e., make simple IO models with varying skin thicknesses)	Has skin that is more realistic than the simple IO model (e.g., skin is textured with pores and hair, different skin colors, etc)
Demonstrates different scenarios/contraindications (e.g., simple IO models with bone fractures, skin infections, etc.)	Has resistance to flow coming either from a syringe or an IV bag (e.g., in the model, incorporate a Luer lock adaptor that you could hook IV tubing up to the back which could stay hidden inside of the model to fill it with red liquid, and then when the IO is established, there would be positive feedback and the ability to run fluids/push meds)
Includes a portion of the calf to help with landmarking (e.g., use a sponge to surround the top of the bone)	Has skin that could show infiltration (e.g., use a bladder pump to show how if you miss the bone during the IO procedure, the space under the skin would fill up with liquid and bubble up)
Made with different densities so that when you insert the needle, it goes in more easily or not? (e.g., make the bone softer for young people, make the bone thinner for 65+, etc.)	Is a full leg
	Shows the muscle/anatomic contours of the leg better than it does now (e.g., create a snug “sleeve” to serve as the skin to slide over the top of the muscle/bone structure of the model)
	Has a bigger patella than what we already have in our model (see the pictures/video above) and be made mobile (e.g., all the other bones in the model are fixed while the patella is made separately to fit in and could give way when you feel for it to help with landmarking).

Results of the modified Delphi methodology

In round one of the modified Delphi methodology, seven informants were sent a survey with the 14 ideas created in the modified design thinking process in the form of questions to rank their importance to include in the simple and advanced IO simulators using Likert scales (Table 2). For each of the ideas, informants were provided an option to add comments, questions, and/or suggestions through free-text fields. In round one of the modified Delphi methodology, all seven informants responded. 

**Table 2:** Modified Delphi round one survey questions IO: Intraosseous, IV: Intravenous

No.	Question
Simple IO Model	
1	How important is it that the simple IO model: Provides resistance when performing the IO technique on it?
3	How important is it that the simple IO model: Simulates the bone marrow? (e.g., in the hollow space of the bone, place a sponge and add liquid to it)
5	How important is it that the simple IO model: Is designed to demonstrate people of different weights (i.e., make simple IO models with varying skin thicknesses)?
7	How important is it that the simple IO model: Is designed to demonstrate different scenarios/contraindications? (e.g., simple IO models with bone fractures, skin infections, etc)
9	How important is it that the simple IO model: Includes a portion of the calf to help with landmarking? (e.g., use a sponge to surround the top of the bone)
11	How important is it that the simple IO model: Is made with different densities so that when you insert the needle, it goes in more easily or not? (e.g., make the bone softer for young people, make the bone thinner for 65+, etc)
13	Please provide any OVERALL comments/suggestions/questions for the simple IO model
Advanced IO Model	
14	How important is it that the advanced IO model: Is able to bend and laterally expose the tibia? (e.g., 360-degree swivel on the superior end so that we can also move the leg and position it on the table)
16	How important is it that the advanced IO model: Is made longer from the knee down?
18	How important is it that the advanced IO model: Has skin that is more realistic than the simple IO model? (e.g., skin is textured with pores and hair, different skin colors, etc)
20	How important is it that the advanced IO model: Has resistance to flow coming either from a syringe or an IV bag? (e.g., in the model, incorporate a Luer lock adaptor that you could hook IV tubing up to the back which could stay hidden inside of the model to fill it with red liquid, and then when the IO is established, there would be positive feedback and the ability to run fluids/push meds)
22	How important is it that the advanced IO model: Has skin that could show infiltration? (e.g., use a bladder pump to show how if you miss the bone during the IO procedure, the space under the skin would fill up with liquid and bubble up)
24	How important is it that the advanced IO model: Is a full leg?
26	How important is it that the advanced IO model: Shows the muscle/anatomic contours of the leg better than it does now? (e.g., create a snug “sleeve” to serve as the skin to slide over the top of the muscle/bone structure of the model)
28	How important is it that the advanced IO model: Has a bigger patella than what we already have in our model (see the pictures/video above) and be made mobile? (e.g., all the other bones in the model are fixed while the patella is made separately to fit in and could give way when you feel for it to help with landmarking).
30	Please provide any OVERALL comments/suggestions/questions for the advanced IO model.

The averages and SDs of these 14 questions along with their comments are provided in Table 3. Two out of 14 questions i.e., questions 14 and 22, reached unanimity (SD of 0.76 and 0.9) amongst the informants. Specifically, they agreed that it is important to update the advanced IO simulator so that it 1) is able to bend and laterally expose the tibia (i.e., have a 360-degree swivel on the superior end so that the leg can be moved and positioned on a flat surface), and 2) has skin that could show infiltration (e.g., use a bladder pump to show how if the bone is missed during the IO procedure, the space under the skin would fill up with liquid and bubble up). Therefore, these questions were deemed resolved for modified Delphi round one, noted as features to include in the updates to the simple IO simulator, and were not revisited for modified Delphi round two. The rest of the questions in the modified Delphi round one scored below the threshold and had high variabilities. Using the commentaries of the informants in the free-text field, these questions either needed revising for further clarification in round two due to mixed opinions or were discarded as the overall notion of the comments suggested that the feature presented in the question was unimportant to include in the updates of the simple IO simulator (Table 3).

**Table 3:** Modified Delphi round 1 average, standard deviations, and comments SD: Standard deviation, IO: Intraosseous, N/A: Not applicable, CE: Centralized education

Question #	Average	SD	Comments
Simple IO Model			
1	4.43	1.13	The IO model must provide strength to simulate real anatomical parts.
			The leg is movable during the procedure and the thigh must rotate externally for proper landmarking and insertion. With the piece being fixed the paramedic will have to adjust their angle to ensure they are inserting at a 90-degree angle.
			There has to be transitional resistance as the needle goes through the skin, then at the bone, then as the needle is advanced through the bone, and finally a loss of resistance when the needle enters the bone matrix to indicate correct placement.
			Very important: this is possibly the most important part of this trainer as the point is to realistically simulate the skill.
			Tactile feel for performing the skill is important for learning.
			I do not think that the simple model requires skin. I feel it is important to understand bone anatomy on its own. The advanced model should incorporate skin, muscle, and subcutaneous tissues.
3	3.71	1.5	Bone marrow is very important. When inserting a needle into a long bone, the contents within the bone marrow must be aspirated and visualized for the procedure to be considered successful.
			I think this provides excellent feedback and is a reminder you may need to hook a pressure infuser up to be able to run fluids rapidly to work against the added pressure.
			This is where the model starts to change. The simple IO model is a good tool for the initiation of the IO. The addition of a sponge/fluid, etc. is nice for the advanced use of the tool in providing fluid or medication administration. It is not required for the initiation.
			Less important, but when developing the muscle memory of the skill it would be ideal. This is often a failure point when performing the skill in real life (forgetting to flush the IO after needle insertion) so the more realistic the better.
			Would be a bonus to have marrow you could draw back but I don't think it's as important as other aspects.
			Many of my successful IO placements have not had positive results for the aspiration of marrow.
5	2.57	1.27	In my experience, the patient's weight does not affect the procedure at all.
			This will allow proper needle selection
			Not really significant to me
			This is less important in my opinion, but the more variety the better. Especially if you can realistically recreate the feeling of proper landmarking.
			This would be a nice added bonus for training and for determining the size of the needle to use, but not essential.
7	2.14	1.07	This would be interesting to expand the content taught and learned.
			I think that is unnecessary
			Less important in my opinion. We must discuss these verbally.
			I don't feel this is an important consideration
9	2.71	1.38	Palpable bony areas are much more important
			Creates more realism
			The landmarking starts from the tibial tuberosity, not the calf. Does it depend on what the model is being created for? If it's just an IO initiation tool, then no. If it is more a part of a full simulation and patient care, then maybe.
			The more realistic and complete the model, the better.
			My first thought with the picture is to extend it to be longer. I think this is important for landmarking and as well for more realistic training.
11	3.14	1.46	This is very important and helps to demonstrate that different ages should be treated in different ways and with different strengths.
			I think different sizes would be better, and more focus on how the pediatric/neonatal bone feels like, recreating the density of that bone. Neonates are the most difficult to landmark with small bones and lots of adipose.
			Bone density will not change that much. The bigger value would be in alternate sizes of IO to emulate infant, child, adolescent, and adult.
			Similar to the "different weight" option - nice, but not a need to have.
			Would be a nice added feature as well.
13	N/A	N/A	Realism is key to success in simulation and learning as well as being able to reuse the same product. A removable insert would be ideal as we have up to 350 medics passing through CE, that’s 350 pokes.
			My main question about the simple vs advanced IO model is that it would be good to know how you see these two different models being used and what the goal is for each model. The most important thing I think from a training perspective is the feel and ensuring that the feel is as realistic as possible. The other stuff is an added bonus if possible to incorporate.
Advanced IO Model			
14	4.29	0.76	I believe the most interesting thing is the flexion of the thigh and not the rotation.
			Being able to actually manipulate the joint would be nice
			This seems like a key design feature to this advanced model.
16	3.86	1.07	I think this would be a waste of time to build and also a waste of material and money. The area around the patella is more than enough to train.
			Maybe nice in a full simulation and advanced IO training, but not required for IO initiation only.
			Same as above - if making an advanced model, it should be as realistic and whole as possible.
			Or insert it into a hollowed-out leg portion on a full leg or mannequin.
18	2.86	1.35	This is cool, and it brings more realism.
			Less important, but would certainly be nice if not too expensive to reproduce.
			I don't think this stuff is necessary but one comment is that the skin should be realistic in that there is generally very thin skin over the tibial area where landmarking occurs so making it anatomically realistic is important.
20	3.71	1.11	Resistance must come from the outside to the inside (saline bag) and not from inside the bone.
			Same as the simple IO model response. It depends on what the tool is being used for.
			I think this would be a good feature
			Probably not important but would be a nice feature
22	4.14	0.9	This would demonstrate a kind of iatrogenic and it would be very interesting if the simulator could simulate this as well.
			Not sure.
			Again a great feature, but not necessary. As an example, you could design the bone to show that it was correctly placed, and the lack of feedback there would show that it is in the incorrect spot.
			I was thinking about this in the simple model - but would make sense that this is a part of the advanced model if it is realistic and reasonable to do.
24	3	1.15	I believe this would be a waste of time and money.
			The more complete the model, the better.
			Not necessary
			Or inserted into a full leg
26	3.57	1.4	Bone palpation is much more important. But OK.
			For an advanced model - the more realistic, the better.
28	3.29	1.5	Having a big or small patella doesn't matter. But it must be palpable enough so that the anatomical area can be recognized by the doctor's hand.
			Patella does not play a role in the landmarking or procedure
			For an advanced model - the more realistic, the better.
30	N/A	N/A	Would be good to add additional features to the advanced model. I think feeling for infiltration in the calf area and some resistance to pushing fluids would be important clinical applications.

Through the comments, some informants strived to determine the appropriate learning objectives of both the simple and advanced IO simulators and offered suggestions. One such idea was that “the simple IO model would be a good tool for the initiation of the IO while the advanced model would be a good tool in providing fluid administration”. Informants agreed that the “loss of resistance” is appropriate to learn from the simple IO simulator as a first step, and then “the aspiration of bone marrow” could be looked at secondarily, possibly in the advanced IO simulator as the next step. The inclusion of the different weights of individuals through varying skin thickness, bone densities according to age, and contradictions (e.g., bone fractures, skin infections, etc.) in the simple IO simulator, and for the advanced IO simulator, the addition of hair, textured skin, and skin colors as well as making the contours of the leg more visible was generally perceived to be insignificant to the procedure but noted to augment the realism. When asked about the lengthening of either the simple IO simulator or the advanced IO simulator beyond the knee to help with landmarking (i.e., either a portion of the calf or a full leg), the informants, again, mentioned that this would also contribute to the realism, but emphasized that the tibial tuberosity is where the landmarking starts and making that region of the simulator palpable would be more important. Finally, for the advanced IO simulator, the average of 3.71 for question 20 indicated that informants agreed that including the resistance to flow coming from either a syringe or IV bag would be a necessary feature to have. However, an SD of 1.11 and the comments for this question suggested heterogeneity in opinions, warranting revising and reexamination of the question in modified Delphi round two. In summary, based on the ratings, distribution of ratings, and comments on the questions in modified Delphi round one, many of the questions needed to be adjusted in order to clarify the updates required to be made to both the simple and advanced IO simulators to increase their effectiveness in teaching the IO access and infusion procedure. 

In round two of the modified Delphi methodology, the same seven informants who participated in modified Delphi round one were sent a survey with a combination of new and revised questions based on the results from modified Delphi round one (Table 4). The modified Delphi round two questions were structured in two different ways. New questions concerning the learning objectives of the simple and advanced IO simulators asked informants to rate their importance using a Likert scale similar to the modified Delphi round one. These new questions were made specifically to obtain consensus on the learning objectives from all the informants as the majority of the informants did not partake in the modified design thinking process to weigh in. As a result, during round one, most of the informants made comments about the learning objectives, not in response to any particular question, suggesting that the learning objectives needed clarification. Based on the thematic analysis conducted on the modified Delphi round one results, some of the comments classified the possible ideas to include as updates to the simple and advanced IO simulators as either “mandatory”, “optional/nice to have”, or “not required”. To confirm what features to include in the simple and advanced IO simulators, all informants were asked to classify the ideas as “mandatory”, “optional/nice to have”, or “not required” through a multiple-choice format in round two. Similar to the modified Delphi round one survey, informants were provided an option to add comments, questions, or suggestions through free-text fields in the modified Delphi round two. In round two of the modified Delphi methodology, all seven informants responded. 

**Table 4:** Modified Delphi round 2 survey questions IO: Intraosseous, IV: Intravenous

No.	Question
Learning Objectives	
1	The simple IO model should focus on teaching the initiation of the IO (i.e., drilling into bone and feeling loss of resistance once the needle enters the bone matrix).
3	The advanced IO model should focus on teaching the aspiration of bone marrow and the administration of fluid.
Simple IO Model	
6	Make the simple IO model with a pronounced tibial tuberosity to facilitate landmarking (more palpable) and to add to the realism
8	Make the simple IO model in different bone sizes to emulate infant, child, adolescent, and adult scenarios
Advanced IO Model	
11	Make the advanced IO model with a pronounced tibial tuberosity to facilitate landmarking (more palpable) and to add to the realism
13	Make the advanced IO model longer from knee down to show full patient care and to add to the realism, but how far? (e.g., to extend to the ankle, including the calf muscles it will increase the price of the model by $200-300)
15	Make the advanced IO model with different skin thicknesses to test for proper needle selection
17	Make the advanced IO model with different bone sizes to emulate infant, child, adolescent, and adult scenarios
19	Make the advanced IO model with skin that has pores, hair, and different skin tones/colors to add to the realism
21	Make the advanced IO model include resistance to flow from an IV bag or syringe to add to the realism
23	Make the advanced IO model with skin that could infiltrate to show that the IO procedure was done incorrectly
25	Make the advanced IO model show muscle/anatomic contours of the leg better to add to the realism

Two questions, questions 1 and 3, in the modified Delphi round two focused on clarifying the learning objectives of the simple and advanced IO simulators (Table 5). Informants agreed that the simple IO simulator should focus on teaching the initiation of the IO (i.e., drilling into bone and feeling loss of resistance once the needle enters the bone matrix) (average of 4.29, SD of 0.95). For the advanced IO simulator, informants overall rated the learning objective of being able to aspirate the bone marrow and administer fluids as important (average of 3.71). However, there was some degree of variability (SD of 1.11) which was reflected in the comments. Informants mostly suggested that the focus of the advanced IO simulator should not be the aspiration of the bone marrow as it is no longer necessary in the procedure but the administration of fluids, particularly honing in on the resistance to flow feature during administration. These comments were used to modify the focus of the advanced IO simulator accordingly. Overall, gauging the learning objectives of the simple and advanced IO simulators through the modified Delphi round two survey, helped with guiding the selection of the features to include in the updates to the simple and advanced IO simulators. 

**Table 5:** Modified Delphi round 2 average, standard deviation, and comments SD: Standard deviation, IO: Intraosseous

Question #	Average	SD	Comments
Learning Objectives			
1	4.29	0.95	Just drilling through the bone without having any content to aspire to in my opinion would train the student just to handle the Gundriver.
			Oddly enough when we teach the skill, we listen and feel for a pop, like it’s breaking through this pressure gradient.
			Landmarking placement is an essential practice that we would include in even the most simple teaching of the skill.
			I think the value in training is from completing the skill. important pieces would be landmarking, initiation, and hooking up iv post-initiation. I gave neutral as I was not sure if you were thinking all of this or just drilling the bone itself. I find less value in drilling the bone if you cannot hook up the IO after - once you have drilled it a few times the usefulness becomes less.
3	3.71	1.11	And the accurate identification of landmarks is proper. Placement.
			This is one of the biggest strengths of a good IO simulator.
			We no longer aspirate marrow for confirmation but definitely for fluid administration especially the delivery of fluid under pressure (pressure infuser attached to the bag that pushes through the resistance) we typically face.
			The advanced IO shouldn't necessarily "focus" on the aspiration aspect of the skill. It should be all-encompassing.
			This model would be great for aspiration, administration, as well as interstitial recognition.
Question #	"Mandatory Optional/Nice to have Not required" (number of votes)		Comments
Simple IO Model			
6	Mandatory (6/7)		This helps to identify the proper location.
			Would make it as anatomically correct as possible - not over-exaggerated but correct anatomy
8	Mandatory (3/7) Optional/Nice to have (4/7)		It helps to have experiences with other types of patients with different ages.
			The silicone will play a huge role when simulating neonate/infant IO Models as the bone-to-fat ratio is very different than an adult.
			Ideally, an adult and an infant would be ideal.
			Would be a nice additional feature
Advanced IO Model			
11	Mandatory (6/7) Not required (1/7)		Different bone shapes and skin types make situations challenging for the user.
			Again, this helps to identify the proper place to insert the needle.
13	Optional/Nice to have (5/7) Not required (2/7)		Suggest having one full-leg model that you can rotate/bend the knee. Interchangeable less expensive models can be inserted and removed as needed.
			I see no reason for this.
			I think it would provide more realism but not necessary
			Could you have both options ultimately? There are arguments on both sides including cost, and portability but also realistically.
			To the knee would be more than enough.
			I guess it depends on the goals of the model. If you want the most realistic model, where you can assess for infiltration, etc. you would require this, which I guess unfortunately would come with a cost. Otherwise, I would say if you are not interested in that degree of realism and you were more concerned about initiation and using the IO, I think you could get away with having enough space that someone could place their other hand below (similar to how you would in real life for stabilization) so I guess in that case 6" below proper landmarking area?
15	Mandatory (3/7) Optional/Nice to have (3/7) Not required (1/7)		There is really only one needle size for infants/children and one needle size for adults. This is independent of skin thickness which is generally not very variable except in the very obese settings.
			The skin over the appropriate needle puncture site is thin. In my opinion, this is not mandatory.
			This is more clear when talking about neonates and infants
			Would be a nice added feature
17	Mandatory (3/7) Optional/Nice to have (4/7)		Being realistic is important and this is for more than the insertion but also rendering treatment and having an appropriately sized bone will add to the realism.
			Different models help to understand different scenarios.
			Would be a nice added training feature.
19	Optional/Nice to have (5/7) Not required (2/7)		In my opinion, this does not add much to the purpose of teaching how to perform this procedure.
			Not sure this adds a ton. It looks neat but in terms of being able to use the model, I am not sure it is as helpful. If it was free it would be a great feature, assuming it costs money I would say that the trade-off may not be worth it.
21	Mandatory (5/7) Optional/Nice to have (1/7) Not required (1/7)		Definitely beneficial
			In my opinion, it's not mandatory but it would be nice to have this.
			Not sure this adds much in terms of usefulness for the skill
23	Mandatory (2/7) Optional/Nice to have (5/7)		As long as there is a way to clean the model after use and remove the infiltrated solution as that is what degrades models most quickly and is also a source of mold growth. Unless the skin is "sealed" to the bone, a missed IO will produce infiltration. The concern would be where the bone is used in more than one IO initiation and therefore there are multiple holes in the bone that then leak. A self-sealing membrane inside the bone matrix would help prevent that.
			This could prove to be very difficult.
			This iatrogenesis happens and is not uncommon. It would be interesting to demonstrate what it looks like and discuss how to avoid it.
			Only if it does not overly complicate the device.
			I think this would be a great feature - something that is currently missing is how to prepare people for this adverse event.
25	Mandatory (1/7) Optional/Nice to have (6/7)		It may be simply the picture but the advanced IO with the skin does not look realistic in the picture. Realism and the ability to properly landmark are crucial.
			This helps to better identify the needle insertion site
			Probably within a certain degree, this would add to the realism although most of the muscle, etc. It is not really involved in any area you start the IO - so not sure it is necessary. If there is a way to have different tissue thicknesses in different areas that correlate to muscle mass, etc., it doesn't matter as much if it is anatomically correct that would allow people to look/feel for the area without tissue.

With regards to the features to include in the updates to the simple and advanced IO simulators, the features that showed high distribution in the ratings during modified Delphi round one, were classified as “mandatory”, “optional/nice to have”, or “not required” by informants in the modified Delphi round two (Table 5). For the simple and advanced IO simulators, most informants ranked having pronounced tibial tuberosity to facilitate landmarking and to add to the realism as a mandatory feature. Another feature that was voted as mandatory by the majority of informants was the demonstration of the resistance to flow from a syringe or an IV bag in the advanced IO simulator. Bone sizes, length of the tibia, skin thicknesses, skin details (i.e., hair, pores, color, texture), muscle contours, and the demonstration of infiltration were all considered “optional/nice to have” features in the simple and advanced IO simulators as they would all add to the realism according to the comments. Using these results from modified Delphi round two, the research team decided to select the features that were chosen as mandatory to include in the simple and advanced IO simulators in order to follow a design-to-cost approach and concluded the modified Delphi methodology.

A summary of the modified design thinking process and the modified Delphi methodology is shown in Table 6. The final output of the modified Delphi round two generated a list of features that would be either mandatory or optional to include in the final designs of the simple and advanced IO simulators. Based on the comments provided by the informants throughout the process, a feature is classified as optional if it adds to the realism of the simulator whereas a feature is classified as mandatory if it adds to the learning objectives of the IO access and infusion procedure. For the simple and advanced IO simulators, the features that were considered mandatory were selected in order to follow a design-to-cost approach. The design-to-cost approach focuses on reducing development costs by sacrificing part of the realism of the model to make the simulator more accessible for learners [17]. A design-to-cost approach was followed in this development of simple and advanced IO simulators to maintain the feasibility of production for training purposes.

**Table 6:** Features of the simple and advanced IO simulators from the modified design thinking process that were selected in each round of the modified Delphi methodology IO: Intraosseous, IV: Intravenous

Design Thinking Results	Delphi Round 1 Results	Delphi Round 2 Results
Simple IO Simulator	Include?	Mandatory?
Provides resistance when performing the IO technique on it	X	X
Simulates the bone marrow (e.g., in the hollow space of the bone, place a sponge and add liquid to it)	X	
Demonstrates people of different weights (i.e., make simple IO models with varying skin thicknesses)		
Demonstrates different scenarios/contraindications (e.g., simple IO models with bone fractures, skin infections, etc.)	X	
Includes a portion of the calf to help with landmarking (e.g., use a sponge to surround the top of the bone)	X	
Made with different densities so that when you insert the needle, it goes in more easily or not? (e.g., make the bone softer for young people, make the bone thinner for 65+, etc.)	X	
Advanced IO Simulator	Include?	Mandatory?
Bends and laterally exposes the tibia (e.g., 360-degree swivel on the superior end so that we can also move the leg and position it on the table)	X	X
Made longer from the knee down	X	
Has skin that is more realistic than the simple IO model (e.g., skin is textured with pores and hair, different skin colors, etc.)	X	
Has resistance to flow coming either from a syringe or an IV bag (e.g., in the model, incorporate a Luer lock adaptor that you could hook IV tubing up to the back side which could stay hidden inside of the model to fill it with red liquid, and then when the IO is established, there would be positive feedback and the ability to run fluids/push meds)	X	X
Has skin that could show infiltration (e.g., use a bladder pump to show how if you miss the bone during the IO procedure, the space under the skin would fill up with liquid and bubble up)	X	
Is a full leg		
Shows the muscle/anatomic contours of the leg better than it does now (e.g., create a snug “sleeve” to serve as the skin to slide over the top of the muscle/bone structure of the model)	X	
Has a bigger patella than what we already have in our model (see the pictures/video above) and be made mobile (e.g., all the other bones in the model are fixed while the patella is made separately to fit in and could give way when you feel for it to help with landmarking).	X	X

Design of the simple and advanced IO simulator 

After the modified Delphi round two, the design ideas for the simple and advanced IO simulators were then relayed to and discussed with the development team, a group of three graduate students from maxSIMhealth, a research laboratory at Ontario Tech University, experienced in 3D digital design and printing and additive manufacturing techniques, in order to determine feasibility from a manufacturing standpoint.

Architecture of the Simple IO Simulator

**Figure 2:**
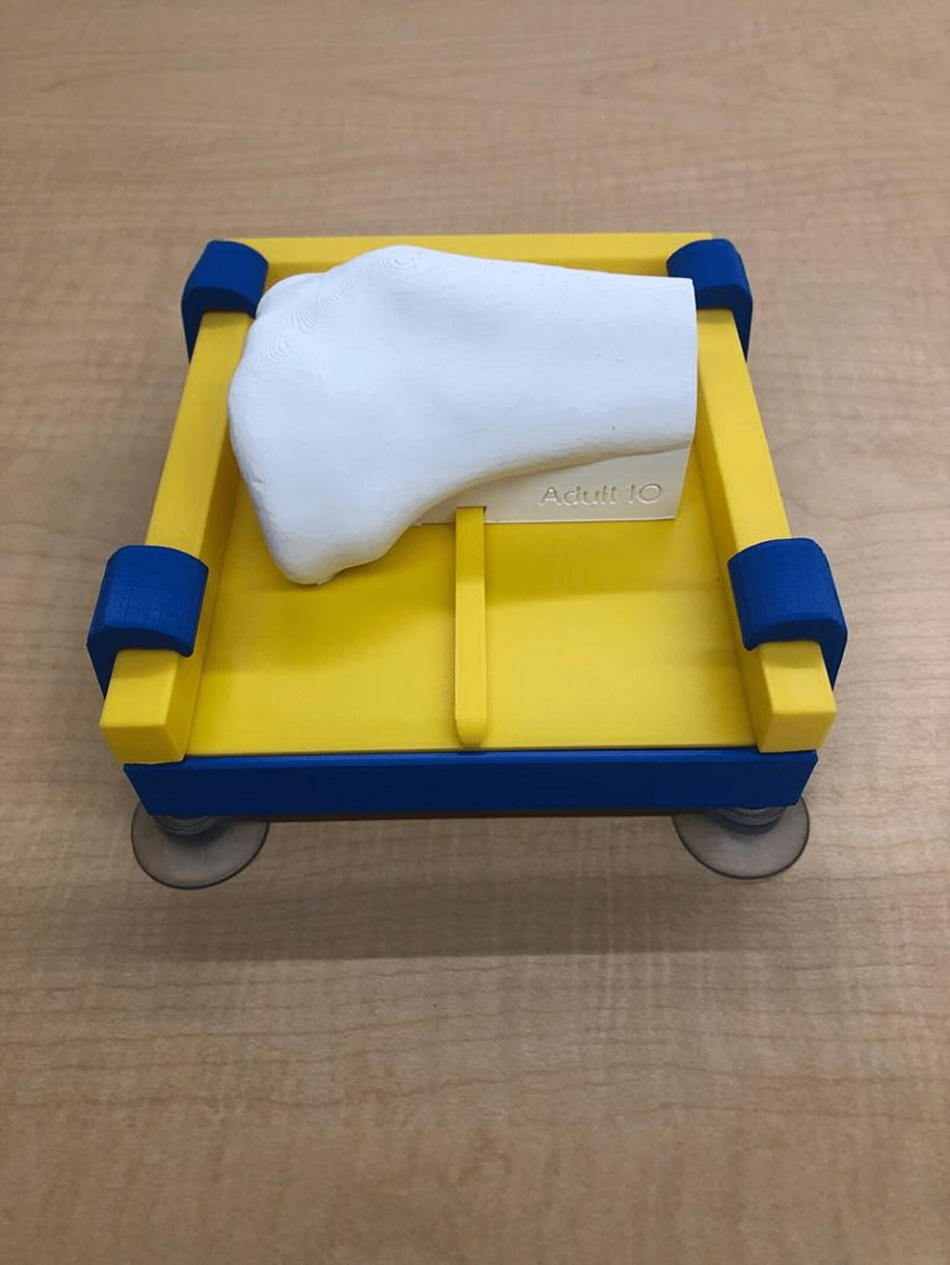
Simple IO simulator on a stand IO: Intraosseous

The simple IO simulator was developed using Fusion360™ (Autodesk Inc., San Rafael, CA, USA), based on the digital assets from a previous project [22]. The digital design was sliced using Ultimaker Cura (Ultimaker B.V., Utrecht, Netherlands), and the parameters were set so that there was a 3 mm wall thickness to better simulate resistance to the drill, and a 30% infill using the line pattern to simulate bone marrow within the tibia. The sliced design was printed using white Ecotough™ polylactic acid (PLA) filament material (Mississauga, Ontario) on an Ultimaker S5 3D printer (Ultimaker B.V., Utrecht, Netherlands). In addition to the bone, a clamp and slide were designed using Fusion360™ so that the bones could friction-fit onto the slide, and be secured onto the stand, as shown in Figure 2. Both parts were also printed using PLA filament material on an Ultimaker S5 3D printer.

**Figure 3:**
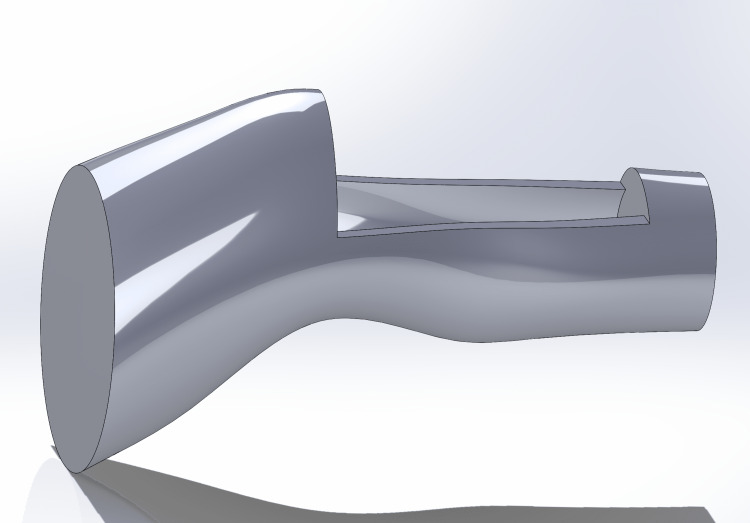
Solidworks rendering of advanced IO simulator with a cut in the top part of the leg that goes from the knee to the middle of the shin to create a small box where the bones can slide in IO: Intraosseous

**Figure 4:**
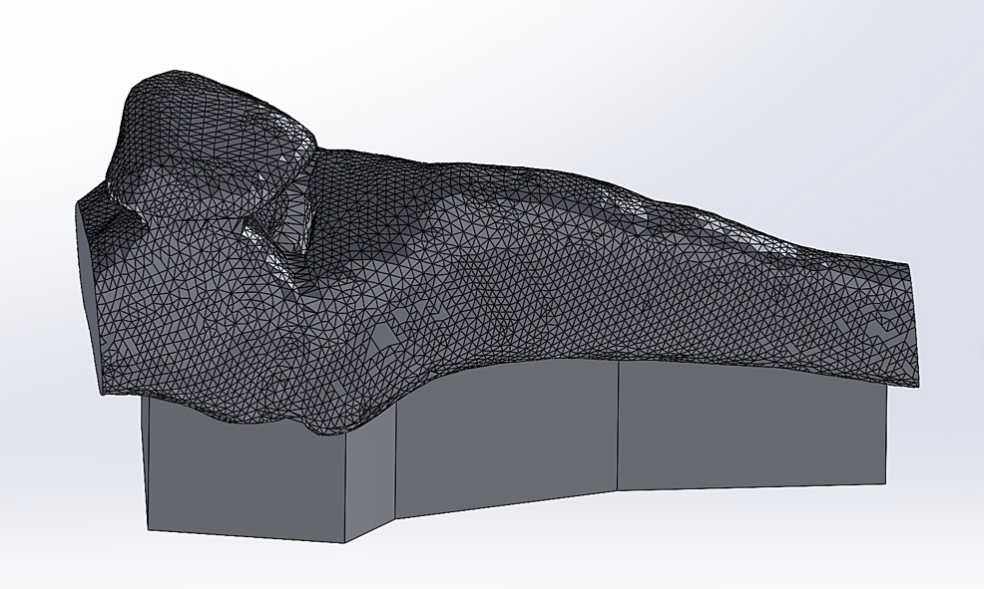
Solidworks rendering of advanced IO simulator modified with a small platform underneath to make the bones more stable at the moment of use IO: Intraosseous

**Figure 5:**
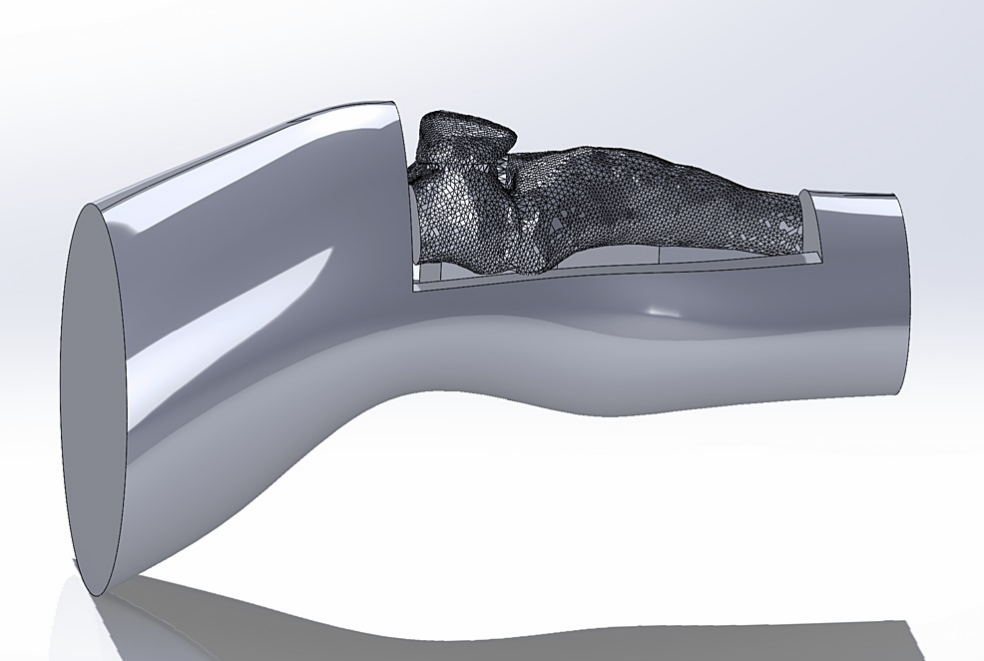
Solidworks rendering of advanced IO simulator showing the leg and the bone insert together IO: Intraosseous

**Figure 6:**
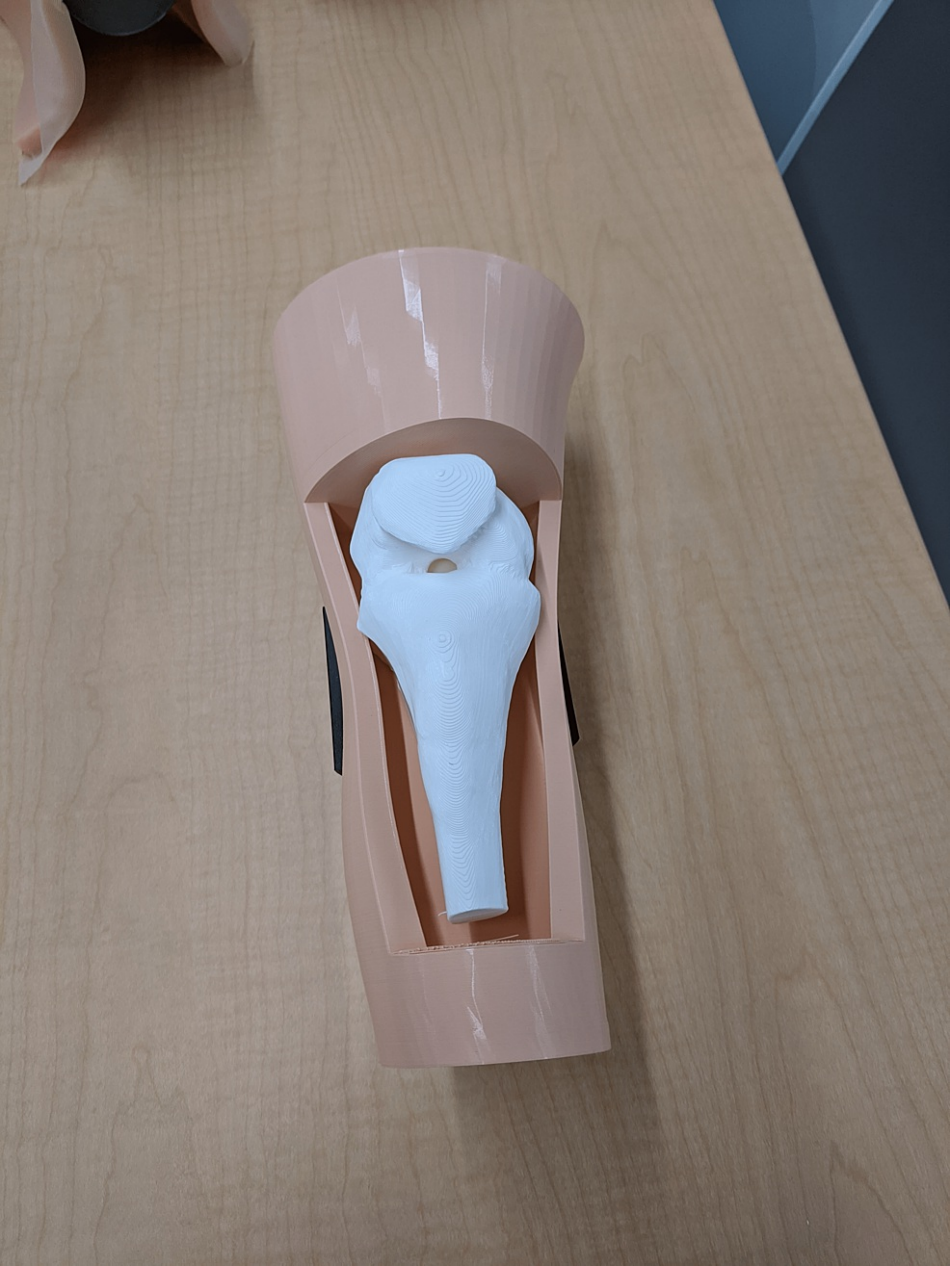
3D-printed advanced IO simulator IO: Intraosseous

**Figure 7:**
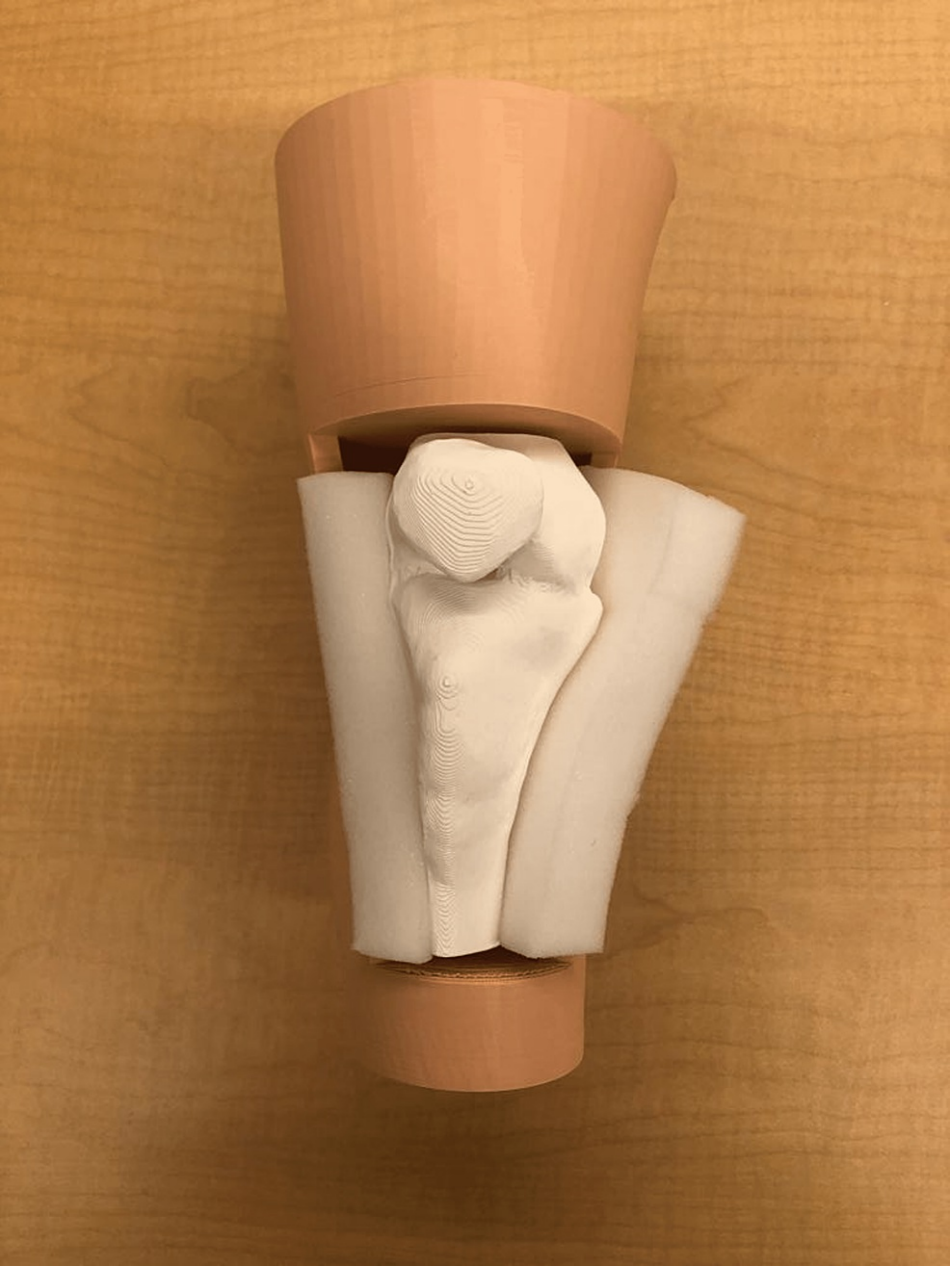
3D-printed advanced IO simulator with foam as fat IO: Intraosseous

**Figure 8:**
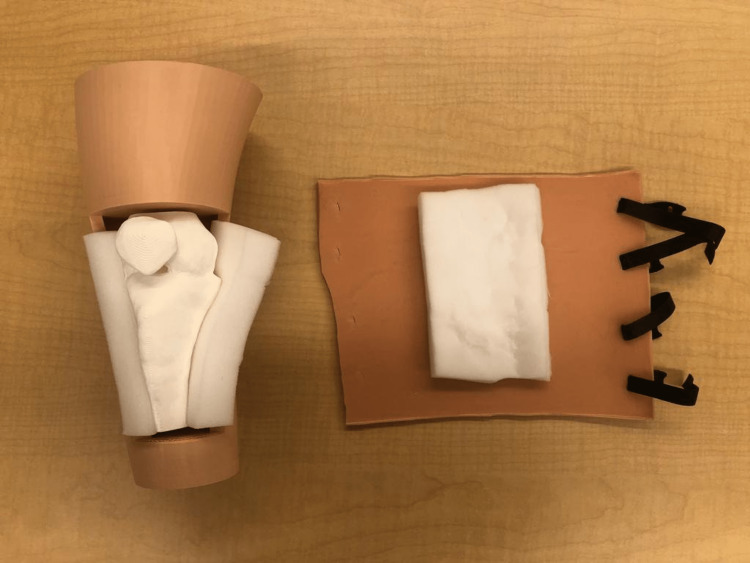
Silicone skin attachment for the 3D-printed advanced IO simulator IO: Intraosseous

**Figure 9:**
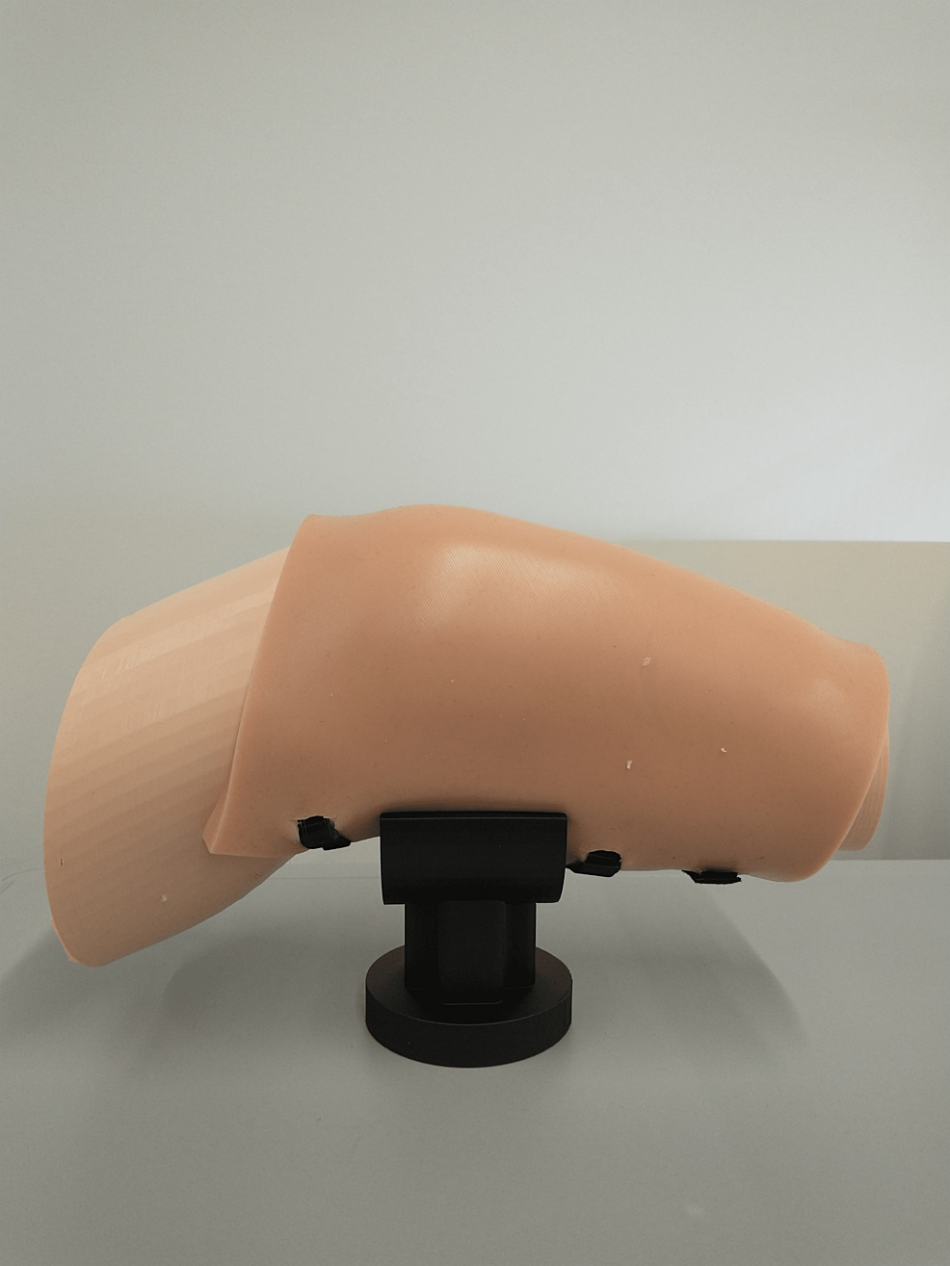
3D-printed advanced IO simulator with silicone skin attachment on a 3D-printed stand IO: Intraosseous

The design process for the IO simulator started based on specific features and adaptations discussed with the informants during the modified design thinking process and modified Delphi methodology to create a more realistic prototype that could fulfill the requirements in a real-life scenario. The proportions and shape of the leg were based on a human leg that was 3D scanned using an Artec Space Spider 3D scanner (Artec3D, Santa Clara, CA, USA) and then customized to 75% of its original size to create a more manageable prototype. The 3D scanned leg was scanned so that the leg is bent at the knee, as requested by the informants. The 3D scanned leg was modified in Solidworks (CAD MicroSolutions Inc ©, Etobicoke, Ontario, CA) to contain a cut in the top part of the leg that goes from the knee to the middle of the shin to create a small box where the bones can slide in, as shown in Figure 3. This leg was 3D printed using skin-tone PLA filament on an Ultimaker S5 3D printer. The bone insert consisted of the patella and the top portion of the tibia. This design was based on available digital assets and was modified using Autodesk Meshmixer (Autodesk Inc., San Rafael, CA, USA) so that a small platform was added underneath to make the bones more stable at the moment of use, as shown in Figure 4. The design for the leg and the bone insert together is shown in Figure 5. The modified designs for the bone were printed using the same 3D slicing parameters as the simple IO bone (3 mm wall thickness and a 30% infill) using white PLA filament on an Ultimaker S5 3D printer. The 3D-printed leg and bone are shown in Figure 6. To simulate a fat layer, a sheet of foam was cut and placed into the empty spaces within the leg surrounding the bone, as shown in Figure 7. To create a skin layer that would wrap around the leg and bone, a rectangular mold was designed in Solidworks, and 3D printed using PLA filament using an Ultimaker S5 3D printer. Ecoflex 00-20 FAST silicone (Smooth-On, Macungie, PA, USA) and Silc-Pig™ coloring skin tone pigments (Smooth-On, Macungie, PA, USA) were combined, poured into the 3D printed mold, set to cure for approximately 75 minutes, and removed to create the skin. Velcro inserts were created along the edges of the silicone skin layer to ensure the skin can be secured tightly around the leg, bone, and foam as shown in Figure 8. Finally, a 3D-printed stand was designed using Solidworks and 3D printed using PLA filament on an Ultimaker S5 3D printer. This stand holds the entire advanced IO simulator so it remains in an upright position, but can still give users the freedom to move if needed (Figure 9).

Costs

Table 7 illustrates the breakdown of all costs associated with the manufacturing of simple and advanced IO simulators. All cost estimates are in Canadian dollars (CAD), including local taxes based on the manufacturing cost of a single simulator.

**Table 7:** Cost breakdown of the materials needed to produce the simple and advanced IO simulators *Includes bone, clamp, and slide. **259g leg + 230g bone insert + 178g (skin mold) ***includes 3 pieces of foam that are 18cm x 8cm=144cm2 **** bone insert (for replacement) is $10.40 from this total (including tax) IO: Intraosseous, CAD: Canadian dollar, PLA: Polylactic acid, N/A: Not applicable

Material	Simple IO Model	Cost of Simple IO Model (CAD)	Advanced IO Model	Cost of Advanced IO Model (CAD)
PLA	280 g*	12.66	667 g**	30.15
Ecoflex™ 00-20 FAST	0	N/A	400 g	22.30
Foam	N/A	N/A	432cm^2***^	1.31
Velcro	N/A	N/A	4 pieces	0.13
TOTAL COST		12.66		53.89****

Strengths, weaknesses, opportunities, and threats (SWOT) analysis survey results

Three of the seven participants who took part in both the modified design thinking process and the modified Delphi study were sent a Google Form survey in the form of a SWOT analysis to provide feedback on the CIC approach [23]. Two participants completed the survey which equates to a 67% response rate. For the strengths of the process, participants stated that the instructions were clear, felt that all ideas could be considered, and the environment was made welcoming and comfortable to share thoughts. Participants also appreciated hearing and viewing the ideas of others, especially those from different scopes of practice (i.e., paramedicine vs medicine), which allowed them to gain insights and build on ideas. With regards to weaknesses, participants indicated that more participants during the design thinking session would have potentially led to further discussion and capturing more ideas. Another area for improvement mentioned was for the design thinking session, where the time for idea generation could have been pushed longer to attempt to force as much volume of ideas as possible so that further content could be included in during the Delphi process. In terms of threats, participants pointed out that technical difficulties using technology to facilitate the process could restrict individuals from fully participating as seen with one of the participants during the design thinking when their microphone stopped working on Google Meet. Lastly, participants highlighted that conducting this process face-to-face with all participants, for the design thinking process as well as after each round of Delphi, could be considered an opportunity as it could allow for further exchange of ideas through group discussions. In sum, this SWOT analysis offered participants’ evaluations of the CIC approach and offered suggestions on where the process could be further improved for future use.

After the survey, an informal interview was conducted with the respondents of the SWOT analysis to pinpoint the feedback for the new approach. The main suggestion that surfaced was that the approach could be used to generate many ideas that could be used in the future. Specifically, during the CIC approach, it was observed that, in addition to providing the details of the elements that were required to construct the simple and advanced IO simulators, the process also led to a list of features that can be employed to change the difficulty of the simple and advanced IO simulators. The features that could achieve this are as follows: 1) demonstrates people of different weights (i.e., make simple IO models with varying skin thicknesses); 2) demonstrates different scenarios/contraindications (e.g., simple IO models with bone fractures, skin infections, etc.); 3) has skin that could show infiltration (e.g., use a bladder pump to show how if you miss the bone during the IO procedure, the space under the skin would fill up with liquid and bubble up); 4) made with different densities so that when you insert the needle it goes in more easily or not (e.g., make the bone softer for young people, make the bone thinner for 65+, etc.).

Features like these are important as modular and customizable simulators can be used as a flexible and adaptive means to challenge learners as they progress through the acquisition of skills. Specifically, the difficulty of the IO skill can be increased by changing the design of the simple and/or advanced IO simulator such as incorporating those features described above. This in turn will lead to the deterioration of immediate practice performance but will lead to improved long-term performance [24]. 

For instance, changes in the thickness of the adipose tissue of the simple or advanced IO simulator can allow the trainee to assess the appropriate IO needle set according to the size and weight of the patient. Another example is to create the bone in the simple or advanced IO simulator in varying sizes and densities so that the trainee can learn how patients of different ages should be treated (i.e., the bone is smaller and softer for younger patients and larger and thinner for older patients and may require less pressure during IO access and infusion compared to adult patients). Different contraindications or scenarios demonstrated in the simple or advanced IO simulator, like tibial fractures and skin infiltrations would challenge the trainee to identify and undertake measures to alleviate and prevent further harm to the patient during the IO access and infusion [25]. By adding these features to the simple and/or advanced IO simulator, the trainee can be exposed to various situations that can increase the complexity of the IO access and infusion procedure, supporting their learning journey.

## Discussion

This original article aimed to describe the development of simple and advanced IO simulators that may be used in the future to enhance the effectiveness of a De-SBE model to teach paramedics-in-training the IO access and infusion skill from any remote location. To accomplish this goal, we designed a unique expert informant crowdsourcing mechanism that consists of a combination of modified design thinking and Delphi methodologies: the CIC approach. 

This approach starts with the articulation of needs and design constraints. In particular, we suggest at minimum to focus on the design-to-cost vs design-to-value approaches. The described test case of development of the simple and advanced IO simulators required a design-to-cost approach. Next, in the ideation phase, we used a design thinking methodology. The advantage of employing design thinking for the development of a simulator is that it generates ideas in a creative and collaborative way. It also fostered a unique collaboration between a university and community healthcare professionals in designing educational tools to advance the care provided by current and future professionals. However, the process lacks the rigor expected by the healthcare simulation community in refining the most important elements that need to be incorporated into the final design. This is often referred to as gathering evidence to support the construct validity of the simulator [19]. Therefore, we substituted the final phases of the design thinking, which narrows down the ideas to a few executable ones, with a Delphi methodology. This method is widely accepted in the simulation community as a tool that provides content validity evidence [20]. 

The use of online tools such as Google Meets and Google Jamboards to facilitate the design thinking process and Google Forms to execute the Delphi study allowed the informants to share their ideas for the design of the simple and advanced IO simulators remotely and instantaneously, contributing to the speed of data collection. The disadvantage to using Google Meets is that it posed technological problems (i.e., Internet connectivity and hardware issues related to audio), and limits individuals from contributing their ideas. The disadvantage to Google Jamboards and Google Forms to collect data on the design of the simple and advanced IO simulators is that information provided by informants can be difficult to interpret and may require follow up whereas, in person, ambiguities could be resolved more readily. Despite these shortcomings, overall, the modified design thinking and modified Delphi methodologies successfully delivered useful information from informants on the design of the IO simulators we aimed to present in this original article. To improve the overall study using these online tools, informants from other geographical areas could be identified and invited to participate in an effort to gather more diverse perspectives and attempt to draw a more generalizable inference. 

Based on the data gathered, we have constructed an affordable advanced IO simulator compared to commercially available trainers. For instance, the Sawbones® (Seattle, USA) Intraosseous Access Injection Trainer is $414 USD [26] while the production costs associated with our simple IO simulator is $12.66 CAD and our advanced IO simulator is $53.89 CAD. In addition, the tibial replacement piece for the Sawbones® trainer is $35.50 USD [27] while the 3D-printing one for our advanced IO simulator is $10.40 CAD. Evidently, the manufacturing of both our simple and advanced IO simulators can be made at a fraction of the costs compared to commercially available trainers. The developmental costs, which include designer time, initial purchases like the 3D printer and its associated consumables (i.e., electricity, parts, maintenance, etc.), have not been added to the production costs, as they have already been covered through research and development funding not applicable to this work.

The stakeholders who tested the CIC approach provided comments about the pros and cons. In general, the features that favor this approach are grouped around the collaborative nature, the clarity of instructions pertaining to the process, and the ability to generate ideas for future use. These features were noted to facilitate the overall design process well with a group of experts. However, they also provided considerations for future CIC approach undertakings, which centered around the use of technology and timing. It was mentioned that although technology allows stakeholders to participate from any remote location, it takes away from rich discussions that could take place in a face-to-face setting. Secondly, the duration of the ideation phase could be given more time to allow for a maximum number of ideas. If these parameters of the CIC approach are adjusted according to the composition of the team involved and the context and construct of the problem in scope, it could result in a better output. 

Finally, following a design-to-cost approach, the development team selected the mandatory features that resulted from the hybrid modified design thinking-Delphi process which allowed us to manufacture the advanced IO simulator at a cost that is a tenth of an average commercially available simulator. The cost was taken into account from the beginning of the design process, with the goal to minimize expenses by surrendering some realism while incorporating features classified as mandatory to include in the final design that would add educational value [28]. At this price tag, these IO simulators are valid, customizable, multifunctional, and inexpensive and as such may be potentially used in the De-SBE model.

## Conclusions

In conclusion, the CIC approach was useful for the purpose of designing a simple and advanced IO simulator that would suit a training plan catered to teach the IO access and infusion procedure decentrally to paramedics-in-training. Specifically, they have been designed in a manner that allows them to be made easily accessible to the trainees i.e., at low costs and high mobility, and work cohesively with online LMSs, which further facilitates the use of a De-SBE model. However, before implementing the De-SBE model within curricula, the development team will test the IO simulators in the DE-SBE model, following the design-based research framework, a process for developing solutions to real-life problems by engaging in iterative design phases and cycles to generate new knowledge and to improve educational practices.
